# Rapid Identification of Major QTL_S_ Associated With Near- Freezing Temperature Tolerance in *Saccharomyces cerevisiae*

**DOI:** 10.3389/fmicb.2018.02110

**Published:** 2018-09-11

**Authors:** Li Feng, He Jia, Yi Qin, Yuyang Song, Shiheng Tao, Yanlin Liu

**Affiliations:** ^1^College of Enology, Northwest A&F University, Yangling, China; ^2^College of Life Sciences and State Key Laboratory of Crop Stress Biology in Arid Areas, Northwest A&F University, Yangling, China

**Keywords:** quantitative trait loci, near-freezing temperature tolerance, *Saccharomyces cerevisiae*, reciprocal hemizygosity analysis, bulk segregant analysis, *NAT1*

## Abstract

Temperatures had a strong effect on many life history traits, including growth, development and reproduction. At near-freezing temperatures (0–4°C), yeast cells could trigger series of biochemical reactions to respond and adapt to the stress, protect them against sever cold and freeze injury. Different *Saccharomyces cerevisiae* strains vary greatly in their ability to grow at near-freezing temperatures. However, the molecular mechanisms that allow yeast cells to sustain this response are not yet fully understood and the genetic basis of tolerance and sensitivity to near-freeze stress remains unclear. Uncovering the genetic determinants of this trait is, therefore, of is of significant interest. In order to investigate the genetic basis that underlies near-freezing temperature tolerance in *S. cerevisiae*, we mapped the major quantitative trait loci (QTLs) using bulk segregant analysis (BSA) in the F_2_ segregant population of two Chinese indigenous *S. cerevisiae* strains with divergent tolerance capability at 4°C. By genome-wide comparison of single-nucleotide polymorphism (SNP) profiles between two bulks of segregants with high and low tolerance to near-freezing temperature, a hot region located on chromosome IV was identified tightly associated with the near-freezing temperature tolerance. The Reciprocal hemizygosity analysis (RHA) and gene deletion was used to validate the genes involved in the trait, showed that the gene *NAT1* plays a role in the near-freezing temperature tolerance. This study improved our understanding of the genetic basis of the variability of near-freezing temperature tolerance in yeasts. The superior allele identified could be used to genetically improve the near-freezing stress adaptation of industrial yeast strains.

## Introduction

Temperature is one of the main relevant environmental variables that microorganisms have to cope with. It has an influence on the life and distribution of nearly all microorganisms. In addition, temperature is also a key factor in some industrial processes involving microorganisms, such as the yeast species (López-Malo et al., 2013). In the yeast *Saccharomyces cerevisiae*, reductions in ambient temperature have several effects on the biochemical and physiological performance of cells: poor protein translation efficiency; slow protein folding; lower membrane fluidity; a reduction in oxygen solubility; changes in lipid composition; changes in nutrient uptake, transport and consumption; an increase in the biosynthesis of some protective compounds; and a reduction in the rate of biochemical reactions (Sahara et al., 2002; Aguilera et al., 2007; Tai et al., 2007; Chiva et al., 2012). The effects of low temperatures on the growth and survival of yeast cells depend on the severity of the stress (Ballester-Tomás et al., 2016). At 10–18°C, metabolic activity and growth rates decrease. When the temperature falls further, near freezing (0–4°C), yeast cells could trigger series of biochemical reactions to respond and adapt to the stress, protect them against sever cold and freeze injury (Ballester-Tomás et al., 2016). It was shown that exposing the yeasts to these severe low temperature prior to freezing enhances the resistance to freezing (Aguilera et al., 2010). The response observed at near-freezing temperatures is more likely to represent the strong cold-shock response for yeasts (Inouye and Phadtare, 2004). However, most studies have focused mainly on the adaption of *S. cerevisiae* at low but still permissive temperatures (10–18°C), there have been limited studies on the temperature below 10°C.

In recent years, the cold shock response at near-freezing temperatures in the yeast *S. cerevisiae* has been studied by genome-wide technologies, including transcriptome and proteomic. Several studies have shown that at these severe low temperatures, accumulation of trehalose, glycerol, and HSPs has found that plays a crucial role in protecting cells from cold or frostbite (Homma et al., 2003; Kandror et al., 2004; Murata et al., 2006; Ballester-Tomás et al., 2016). Different *S. cerevisiae* strains vary greatly in their ability to grow at near-freezing temperatures (Murata et al., 2006; Aguilera et al., 2010). In spite of the recent advances made, the molecular mechanisms that allow yeast cells to sustain this response are not yet fully understood, and the genetic basis of tolerance and sensitivity to near-freeze stress remains unclear.

As with many stress-resistance traits, near-freezing stress tolerance shares the common features of quantitative traits, i.e., multiple genes control and environmental impact. QTL mapping is the main method to dissect the genetic architecture of quantitative traits, and has been successfully applied to various stress-resistance traits in *S. cerevisiae*, such as ethanol tolerance (Hu et al., 2007; Swinnen et al., 2012; Pais et al., 2013), thermotolerance (Sinha et al., 2008; Marullo et al., 2009; Parts et al., 2011; Yang et al., 2013; Wilkening et al., 2014), acetic acid tolerance (Geng et al., 2016; Meijnen et al., 2016), dehydration stress tolerance (López-Martínez et al., 2015), some drugs tolerance (Ehrenreich et al., 2010; Wilkening et al., 2014), and low-temperature tolerance (García-Ríos et al., 2017). Compared with the traditional QTL analysis strategy by constructing the genetic map, bulked-segregant analysis (BSA) can identify the major genes or QTLs rapidly by genotyping only two bulked populations with distinct or opposite extreme phenotypes, is a simple and cost-effective mapping approach (Michelmore et al., 1991). The recent development of whole genome high-throughput sequencing technologies, which can be used to simultaneously measure the allele frequencies in each bulk, has made BSA more efficient, accelerated the QTLs analysis of the yeast *S. cerevisiae* (Ehrenreich et al., 2010; Hubmann et al., 2013a).

In the present study, to investigate the genetic basis of near-freezing temperature tolerance in *S. cerevisiae*, we constructed a recombined F_2_ segregant population by crossing two Chinese indigenous *S. cerevisiae* strains with a divergent phenotype and used the BSA to identify major QTLs involved in the near-freezing temperature tolerance. Two bulks of segregant (21 and 23 individuals respectively) with distinct extreme phenotypes were constructed and whole-genome resequencing analysis was carried out. Finally, a region on chromosome IV was identified tightly associated with the near-freezing temperature tolerance. Reciprocal hemizygosity analysis (RHA) was also used to validate the causative genes within the region. This study improved our understanding of the genetic determinants of variation in near-freezing temperature tolerance in yeast.

## Materials and methods

### Main strains and medium

The main *S. cerevisiae* strains used are listed in Table 1. The YPD medium containing 10 g/L yeast extract, 20 g/L peptone, 20 g/L glucose, solidified with 20 g/L agar when required, was used for the genomic DNA extraction and phenotypic evaluation experiments. G418 (100 mg/L), hygromycin B (300 mg/L), or phleomycin (20 mg/L) was added to this medium for the selection of strains with *KanMX, HphMX*, or *ble*^*r*^ dominant drug-resistance markers, respectively. SD-Ura and SD-Lys-Ura mediums (Solarbio Life Sciences Co., Ltd., China) was used for selecting the auxotrophic strains. ACK medium (10 g/L potassium acetate, 2 g/L agar) was used for sporulation experiments. *E. coli* strain DH5α was used for amplification of plasmids (pUG6, pUG66, and pYC140). This strain was grown in Luria-Bertani (LB) medium (5 g/L yeast extract, 10 g/L tryptone, 10 g/L NaCl, pH 7.5) at 37°C.

**Table 1:** The main *S. cerevisiae* strains used in this study.

**Strain**	**Description**	**Source**
ZX11	Diploid, Chinese indigenous *S. cerevisiae* strain	College of Enology in Northwest A&F University
NX9	Diploid, Chinese indigenous *S. cerevisiae* strain	College of Enology in Northwest A&F University
ZX11(6)	Haploid segregant from ZX11, Matα	This study
NX9(4)	Haploid segregant from NX9, Mata	This study
ZX11(6)/NX9(4)	Hybrids diploid strain obtained by crossing ZX11(6) and NX9(4)	This study
ZX11(6)/NX9(4) *bre1*Δ	Hybrids diploid strain; ZX11(6) crossed with NX9(4) *bre1*Δ	This study
ZX11(6) *bre1*Δ/NX9(4)	Hybrids diploid strain; ZX11(6) *bre1*Δ crossed with NX9(4)	This study
ZX11(6)/ NX9(4) *bdf2*Δ	Hybrids diploid strain; ZX11(6) crossed with NX9(4) *bdf2*Δ	This study
ZX11(6) *bdf2*Δ/NX9(4)	Hybrids diploid strain; ZX11(6) *bdf2*Δ crossed with NX9(4)	This study
ZX11(6)/NX9(4) *nat1*Δ	Hybrids diploid strain; ZX11(6) crossed with NX9(4) *nat1*Δ	This study
ZX11(6) *nat1*Δ/NX9(4)	Hybrids diploid strain; ZX11(6) *nat1*Δ crossed with NX9(4)	This study
ZX11(6)/NX9(4) *cbs1*Δ	Hybrids diploid strain; ZX11(6) crossed with NX9(4) *cbs1*Δ	This study
ZX11(6) *cbs1*Δ/NX9(4)	Hybrids diploid strain; ZX11(6) *cbs1*Δ crossed with NX9(4)	This study
ZX11(6)/NX9(4) *slc1*Δ	Hybrids diploid strain; ZX11(6) crossed with NX9(4) *slc1*Δ	This study
ZX11(6) *slc1*Δ/NX9(4)	Hybrids diploid strain; ZX11(6) *slc1*Δ crossed with NX9(4)	This study
ZX11(6) *nat1*Δ/NX9(4) *nat1*Δ	Hybrids diploid strain; ZX11(6) *nat1*Δ crossed with NX9(4) *nat1*Δ	This study
NX9 *nat1*Δ/*nat1*Δ	Diploid strain; both *NAT1* alleles were deleted	This study
NX9 *NAT1*^ZX11(6)^/ *NAT1*^ZX11(6)^	Diploid strain; both *NAT1* alleles were replaced by the *NAT1^*ZX*11(6)^*	This study

### Phenotypic identification of low-temperature tolerance

Four degree celsius was taken as the reference temperature for near-freezing temperature and 28°C was used as the optimum reference temperature. The near-freezing temperature tolerance of *S. cerevisiae* strains was characterized by the maximum specific growth rate or spot growth on plates. Growth of yeast strains was monitored by turbidimetry determining the optical density (OD) at 600 nm. At 28°C, measurements were conducted hourly for 3 days after 20 s pre-shaking. At low-temperature (4°C) however, microplates were incubated outside and then placed inside the spectrophotometer before being measured. The measurements were taken every 8 h for 18 days. Microplate wells were filled with 0.25 mL YPD medium to ensure an initial OD of approximately 0.1. The uninoculated wells of each experimental series were also determined, to subtract the noise signal. All experiments were performed in triplicate.

Biological growth parameters were deduced from each treatment by directly fitting OD measurements vs. time to the reparameterized Gompertz equation proposed by Zwietering et al. (1990):

$$\begin{array}{l} \text{y}={\text{D}}^{*}\exp\left\{-\exp\left[\frac{{\text{μ}}_{\max}^{*}\text{e}}{\text{D}}\left(\text{λ}-\text{t}\right)+1\right]\right\} \end{array}$$

where y = ln(OD_t_/OD_0_), OD_0_ is the initial OD and OD_t_ is the OD at time t; D = ln(OD_∞_/OD_0_) is the OD value reached with OD_∞_ as the asymptotic maximum, μ_max_ is the maximum specific growth rate (h^−1^), and λ is the lag phase period (h).

Plate spot assay was carried out using the methods reported by Yang et al. (2013) with some modifications. *S. cerevisiae* strains were inoculated in 5 mL YPD medium and grown at 28°C for 24 h. Then, 200 μL pre-culture were inoculated in 5 mL YPD and cultured at 28°C until the exponential phase and were adjusted to OD_600_ = 1.0. Afterward, serial dilutions (1–10^−3^) of the cell cultures were spotted (5 μL) onto YPD agar plates, incubated at 28°C for 3 days or at 4°C for 18 days, respectively. All spot tests were repeated thrice, starting with independent cultures.

### Construction of the haploid parental strains

One copy of the *HO* gene was disrupted by inserting *KanMX* cassette from pUG6 in diploid strains ZX11 and NX9. Then they were sporulated and 20 haploid *ho* spores were collected, respectively. All spores were tested for their growth at 4°C, and two haploids ZX11(6) and NX9(4) were selected as parental strains. The *URA3* gene in ZX11(6) and NX9(4) were deleted by inserting *ble*^*r*^ gene, which amplified from plasmid pUG66 by PCR. Yeast was transformed with the LiAc/PEG method (Gietz et al., 1995) and transformants were selected on YPD medium containing 20 μg/mL phleomycin. They were also unable to grow in the absence of uracil. The presence of the deletion cassette was verified by PCR. Next, the *LYS2* gene was replaced with the *URA3* gene in the NX9(4) strain. Transformants were selected on SD-Ura plates and the correct insertion was also verified by PCR. Finally, two haploid parents strain ZX11(6) (*MAT*α*, ho::KanMX, ura3::ble*^*r*^) and NX9(4) (*MATa, ho::KanMX, ura3::ble*^*r*^, *lys2::URA3*) were constructed successfully.

### Construction of the F_2_ segregant population and two pools

ZX11(6) (*MAT*α, *ho::KanMX, ura3::ble*^*r*^) and NX9(4) (*MATa, ho::KanMX, ura3::bler*, l*ys2::URA3*) were crossed in YPD plates and grown overnight. Patches were replica plated in SD-Lys-Ura plates to select for diploid F1 hybrid ZX11(6)/NX9(4). F1 hybrid was then sporulated and the spores were collected, generated the F_2_ segregant population. The growth at 4°C of all F_2_ segregants was determined. Afterward, two pools were made by selecting extreme individuals from the F_2_ population segregants with the basic statistics of the phenotypic data. The superior segregants (displaying high tolerance to near-freezing temperature) were assembled in the “High-tolerance pool” while inferior segregants (displaying low tolerance to near-freezing temperature) were used to assemble the “Low-tolerance pool.”

### Mating, sporulation, and spore isolation

Mating, sporulation and spores isolation were performed according to Parts et al. (2011). The cultures were grown overnight and plated on ACK medium at 23°C to be sporulated. When the sporulation efficiency has reached >90%, cells were collected and resuspended in sterile water and treated with an equal amount of ether to kill unsporulated cells. Then, cells were washed in sterile water for 4 times, resuspended in 900 μL of sterile water and treated with Zymolase (10 mg/mL) to remove the ascus. After that, mixtures were vortexed for 5 min and diluted to obtain single colonies on plates. The YPD plates containing 100 μg/mL G418 were incubated at 28°C for 3 days and the colonies were collected. Single colonies were re-plated and tested for mating type. The mating type was determined by diagnostic PCR for the MAT locus (Huxley et al., 1990).

### Preparation of DNA samples

The two parent strains ZX11(6), NX9(4), as well as 21 superior segregants and 23 inferior segregants were individually inoculated in 30 mL YPD medium and grown to the stationary phase at 28°C. The genomic DNA was extracted using the methods described by Jubany et al. (2008). The concentration and quality of DNA samples were estimated with a Nanodrop 3000 UV-Vis spectrophotometer (Wilmington, DE, USA). Two DNA pools were prepared by individual genomic DNA extraction and pooling the DNA in equimolar concentrations. Genomic DNA of two pools and both parents were prepared for the following sequencing.

### Pooled-segregant whole-genome sequence analysis and QTL mapping

At least 5 μg of the genomic DNA was provided to Novogene Bioinformatics Technology Co.Ltd. (Beijing, China) for whole-genome sequencing using the illumina HiSeq 4000 platform. Paired-end short treads of ~150 bp were generated and aligned to the genome sequence of the S288c reference strain. BWA (Burrows-Wheeler Aligner) (Li and Durbin, 2009) was used to align the clean reads of each sample against the reference genome. Alignment files were converted to BAM files using SAMtools software (Li et al., 2009). In addition, potential PCR duplications were removed using SAMtools command “rmdup.” If multiple read pairs have identical external coordinates, only retain the pair with the highest mapping quality. SNP calling was performed using GATK v3.3-0 software, with default parameters. QTL analysis based on the distribution of SNP-index and Δ(SNP-index) over the chromosomes was performed according to Takagi et al. (2013). SNP-index was calculated by dividing the number of the alternative variant by the total number of aligned reads. Using NX9(4) as reference genome, SNP-index value was 0 when all short reads contain genomic fragments from NX9(4) and SNP-index value was 1 when all short reads contain genomic fragments from the other parent ZX11(6). If the SNP-index value was <0.3 in both pools, this indicated that false SNPs were caused by sequencing or alignment errors. Those SNPs could not be distinguished and were filtered out. However, some SNPs were filtered with an SNP-index value that was less or more than 0.3 in only one of two bulks and such cases it was assumed that these were putative SNPs (Takagi et al., 2013). Next, Δ(SNP-index) of each filtered SNP was calculated. The difference between SNP-index of “High-tolerance pool” and that of “Low-tolerance pool” was calculated as the Δ(SNP-index). A very high Δ(SNP-index) was indicative of a one-sided SNP preferentially inherited from the superior parent, indicating a genetic linkage to high tolerance to near-freezing temperature. In addition, according to the SNP density, a sliding window analysis with 50 kb window size and 200 bp increment was used for the calculation of the average Δ(SNP-index) of SNPs located in a certain genomic interval. Δ(SNP-index) graph was plotted by aligning an average Δ(SNP-index) against the position of each sliding window in the genome. The top 1% intervals with the highest Δ(SNP-index) values was used as the threshold for selection of the candidate regions.

### General molecular biology techniques

Genomic DNA extraction was carried out as described by Jubany et al. (2008). The rTaq DNA polymerase [Takara Biomedical Technology (Beijing) Co., Ltd., China] was used for diagnostic purposes and Phanta® super-Fidelity DNA polymerase (Vazyme Biotech Co, Ltd., China) was used for amplification of insertion cassettes and sequencing. PCR reactions with the two polymerases were both performed according to the manufacturer's procedure. Gene deletions were carried out using the single-step PCR gene deletion method reported by Wach et al. (1994). Yeast cells were transformed using LiAc/PEG method (Gietz et al., 1995).

### Reciprocal hemizygosity analysis (RHA)

Genomic intervals of the QTL_S_ were analyzed in *Saccharomyces* Genome Database (SGD) (Wang et al., 2013). DNA sequences of candidate genes were downloaded from the NCBI GenBank database. To validate the candidate genes, RHA was carried out. It was performed as previously described by Steinmetz et al. (2002) in the diploid ZX11(6)/ NX9(4) genetic background. Two types diploids were constructed by crossing ZX11(6) and NX9(4) wild type or deletion strains for the candidate gene, so that the resulting hybrid diploids only carried a single allele of candidate gene being evaluated from either ZX11(6) or NX9(4). Deletion cassettes for candidate gene were constructed using the hygromycin resistance marker *HphMX* amplified from pYC140. Transformants were selected on YPD medium containing 300 μg/L Hygromycin B. A specific PCR was carried out to check that the gene was deleted. Low-temperature growth assay of all reciprocal hemizygotes was performed by plating serial dilutions of cells onto YPD plates. The hybrid diploid ZX11(6)/ NX9(4) was added as a control.

### *NAT1* allele replacement in strain NX9

The *NAT1* allele of ZX11(6) was inserted twice in the original sensitive parent strain (NX9), which had both *NAT 1* alleles deleted according to Akada et al. (2002). The both *NAT1* alleles deleted strain was constructed by introducing a disruption cassette flanked with *loxP* sites using homologous recombination (Gueldener et al., 2002). Selectable marker was removed using the Cre recombinase twice. Two *NAT1*_ZX11(6)_ insertion cassettes, *P1*-*loxP*-*AMD1*-*loxP*-*NAT1*^ZX11(6)^ and *P1*-*ble*^*r*^-*GIN11*-*NAT1*^ZX11(6)^, were transformed successively. After the first transformation of the *P1*-*loxP*-*AMD1*-*loxP*-*NAT1*^ZX11(6)^ cassette, positive colonies were selected on the acetamide medium. The correct integration of the insertion cassette was verified by PCR. In the second transformation, the *P1*-*ble*^*r*^-*GIN11*-*NAT1*^ZX11(6)^ was transferred. Positive clones were selected on 20 μg/mL phleomycin containing acetamide medium. Correct integration was also verified by PCR.

### Data access

All sequence data have been deposited in the Sequence Read Archive (SRA) at the National Center for Biotechnology Information (NCBI), http://www.ncbi.nlm.nih.gov/sra, with the account number SRP139301.

## Results

### Screening of parent strains for genetic mapping

**Figure 1:**
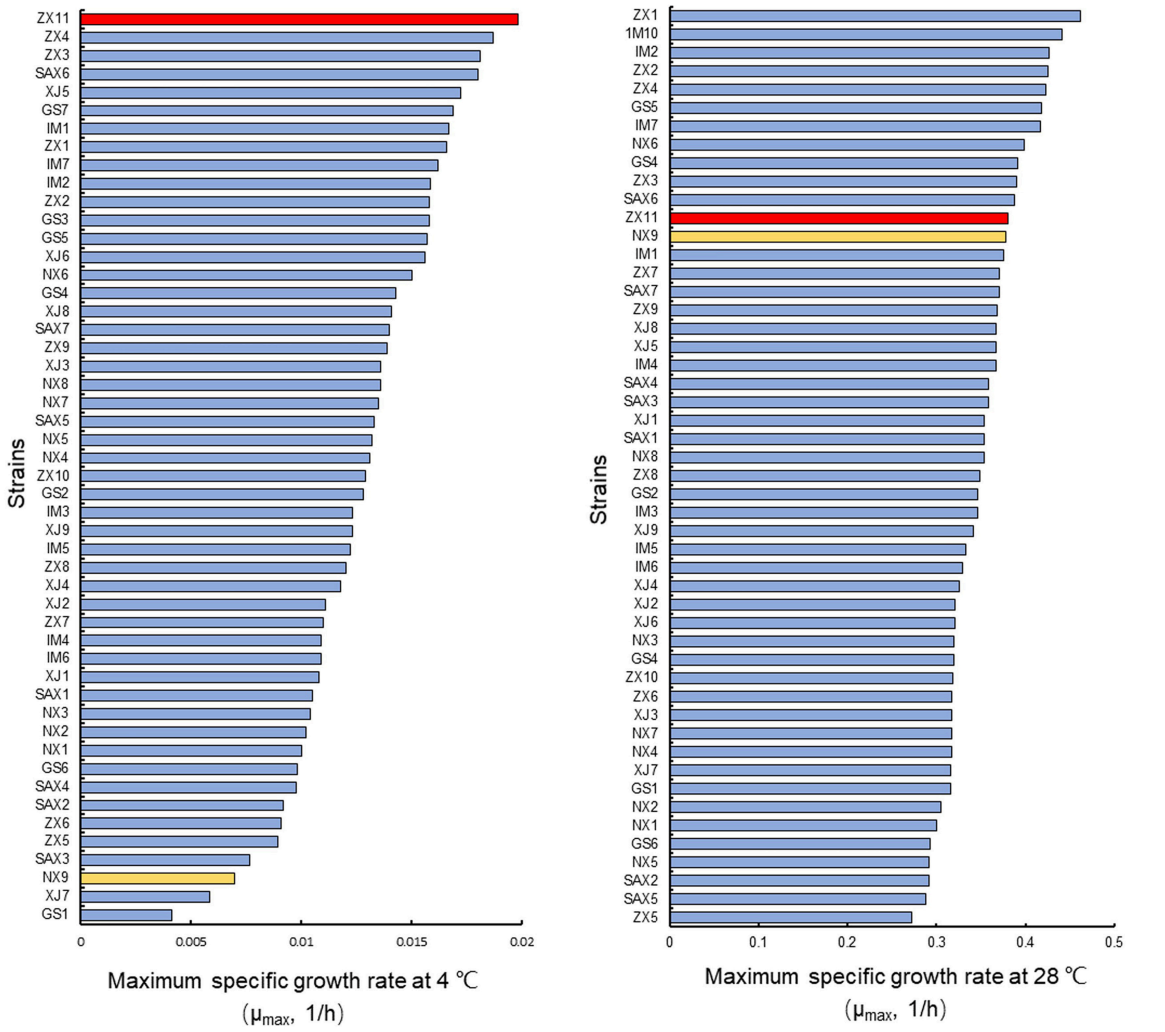
Selection of strains with evident different growth performance at low-temperature. Strain ZX11 (red) and NX9 (yellow) were selected on the basis of the μ_max_ values in YPD at 4 and 28°C. The strain ZX11 exhibited the best growth performance at 4°C of all strains tested. Strain NX9 was ranked among the strains with lower μ_max_ values at 4°C. However, there was no significant difference in their growth performance at 28°C. Hence, ZX11 was selected as the superior strain, NX9 was selected as the inferior strain.

All 50 wild diploid *S. cerevisiae* strains (Supplementary Table S1) from different origins were evaluated for their ability to grow at 4°C and the optimum temperature (28°C) (Figure 1). The μ_max_ values were used to select two diploid strains with evident different growth performance at 4°C, but with no significant differences at 28°C. The strain ZX11 exhibited the best growth performance at 4°C of all strains tested. Strain NX9 was ranked among the strains with lower μ_max_ values at 4°C. However, there was no significant difference in their growth performance at 28°C. Therefore, ZX11 was used as the superior strain, NX9 was chosen as the inferior strain.

**Figure 2:**
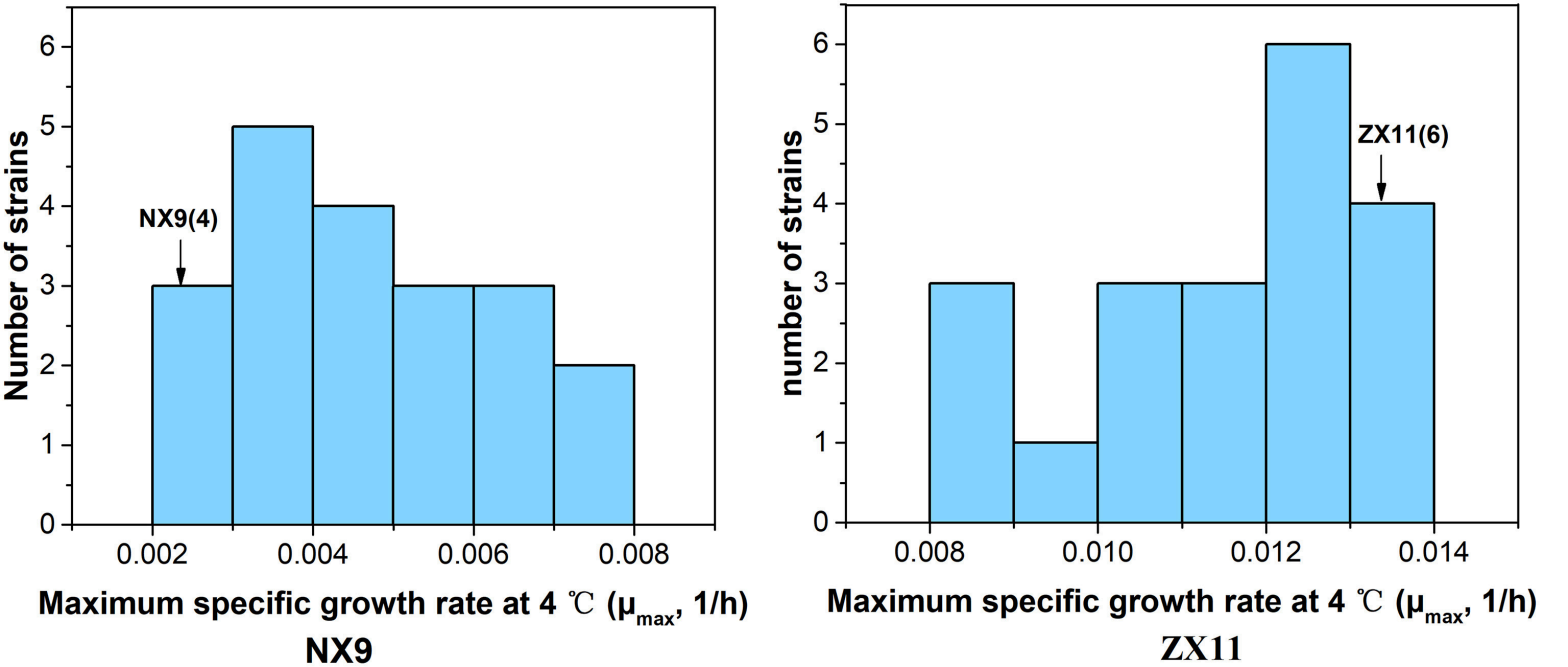
The distribution of the μ_max_ in the haploid *ho* segregants of ZX11 and NX9 at 4°C. The ZX11 segregant, ZX11(6), that displayed the highest μ_max_ value (0.0132) in all segregants at 4°C, was chosen as the superior parent strain. A NX9 segregant, NX9(4) only grew slightly with the μ_max_ value of 0.0025, was used as the inferior parent strain.

**Figure 3:**
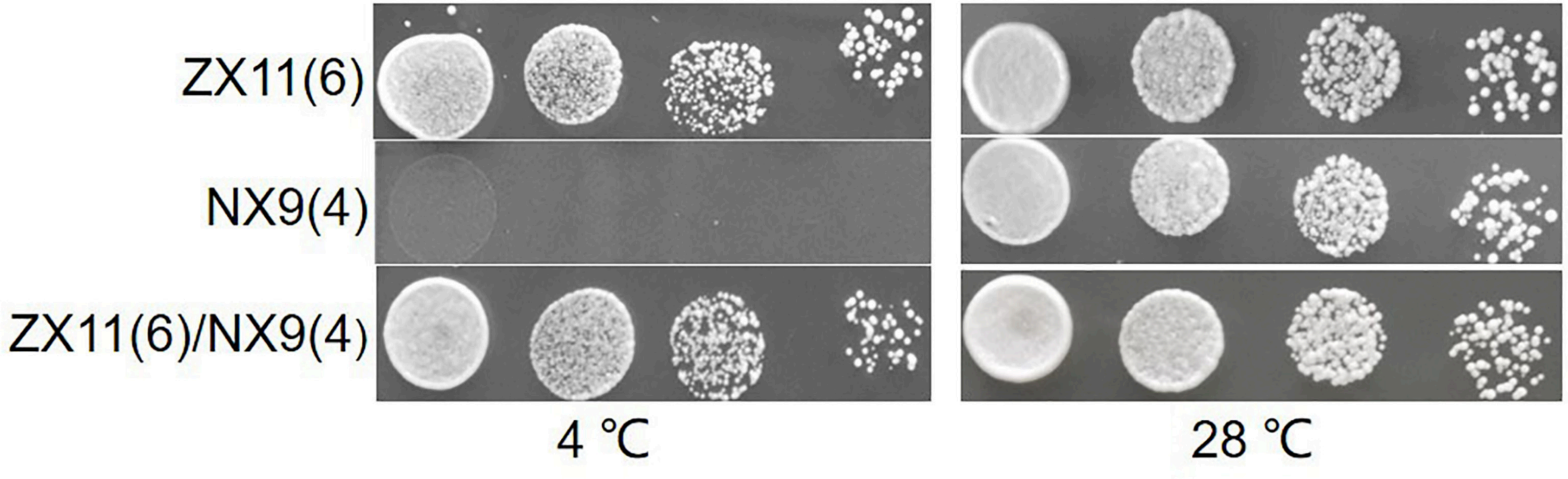
Spot growth at near-freezing temperature of the two parent strains and F1 hybrid. The growth on plates at 4 and 28°C of ZX11(6), NX9(4), and ZX11(6)/NX9(4) were shown. The haploid strain ZX11(6) showed excellent growth at 4°C compared to the strain NX9(4). The hybrid diploid strain ZX11(6)/NX9(4) grew nearly as well as its superior parent strain ZX11(6) at 4°C, indicating that the major causative allele(s) in ZX11(6) is (are) dominant.

To obtain stable haploids for QTL mapping, one copy of the *HO* gene was disrupted by inserting *KanMX* cassettes in ZX11 and NX9. Then they were sporulated and 20 haploid *ho* segregants of each diploid were collected. The distribution of the μ_max_ in the haploid *ho* segregants of ZX11 and NX9 at 4°C were shown in Figure 2. The ZX11 segregant, ZX11(6) (*MAT*α), that displayed the highest μ_max_ value (0.0132) in all segregants at 4°C, was chosen as the superior parent strain. A NX9 segregant, NX9(4) (*MATa*) only grew slightly with the μ_max_ value of 0.0025, was used as the inferior parent strain. ZX11(6) *(MAT*α, *ho::KanMX, ura3::ble*^*r*^) and NX9(4) (*MATa, ho::KanMX, ura3::ble*^*r*^, *lys2::URA3*) were constructed and crossed with each other to obtain the F1 hybrid ZX11(6)/NX9(4). Spot tests of them were also performed and the results were shown in Figure 3. The ZX11(6)/NX9(4) displayed a similar tolerance to near-freezing temperature as the ZX11(6) parent strain, indicating that the near-freezing temperatures tolerance in this strain is a dominant characteristic.

### Selection of the two pools of segregants with extreme phenotypes

**Figure 4:**
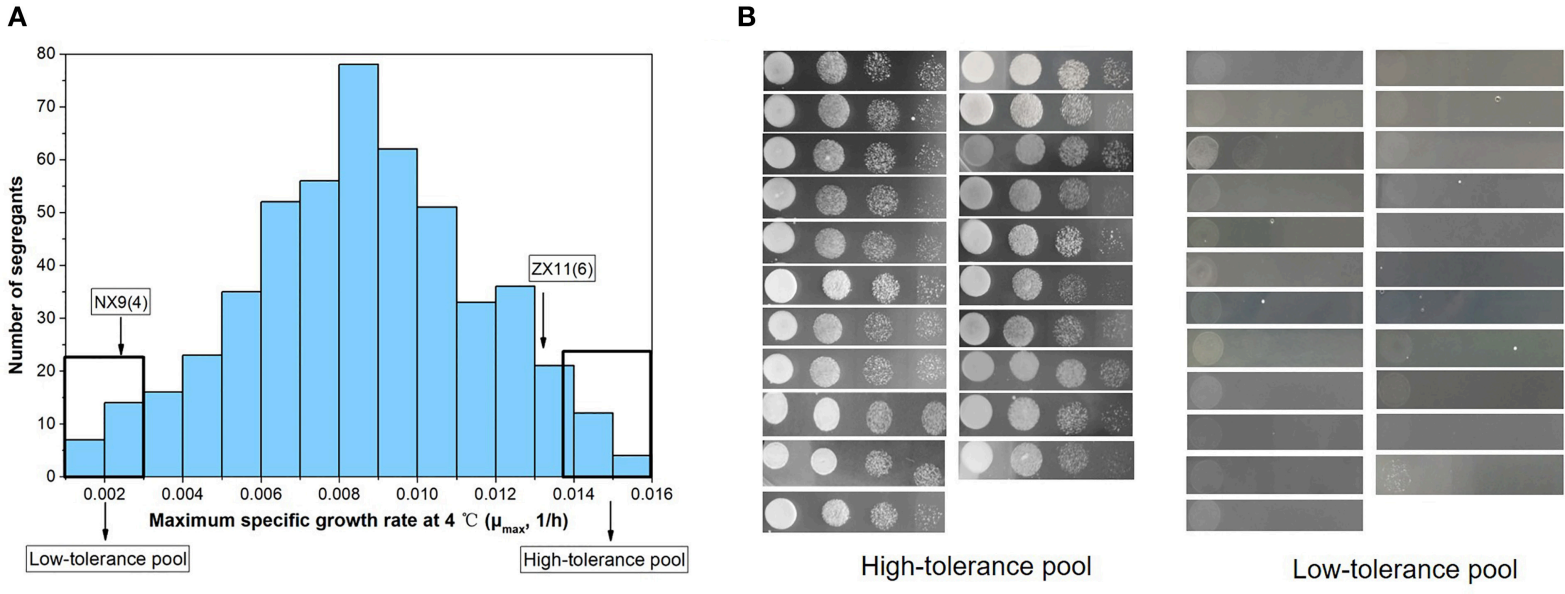
The distribution of the μ_max_ in the F_2_ population segregants and two extreme pools were selected for sequencing. **(A)** The distribution of the μ_max_ in 500 haploid segregants from ZX11(6)/NX9(4). The continuous variation as well as normal frequency distribution was shown. Twenty-one superior segregants with higher μ_max_ (>0.0135) were assembled in the “High-tolerance pool” while 23 inferior segregants with lower μ_max_ (< 0.0030) were selected to assemble the “Low-tolerance pool.” **(B)** Spot growth on plates at 4°C of the superior segregants and inferior segregants.

The F1 hybrid ZX11(6)/NX9(4) were sporulated and about 500 F_2_ haploid segregants were isolated and tested for their growth at 4°C. The distribution of the μ_max_ in the 500 haploid segregants were shown in Figure 4A. The continuous variation as well as normal frequency distribution of μ_max_ in the haploid segregants from ZX11(6)/NX9(4) was apparent, indicating that near-freezing temperature tolerance is a quantitative trait. Among them, 21 superior segregants displaying high tolerance to near-freezing temperature (μ_max_ > 0.0135) were assembled in the “High-tolerance pool” while 23 inferior segregants displaying low tolerance to near-freezing temperature (μ_max_ < 0.0030) were selected to assemble the “Low-tolerance pool.” Spot assay of the superior segregants and inferior segregants were also performed (Figure 4B).

### Identification of QTLs by pooled-segregant whole-genome resequence analysis

**Figure 5:**
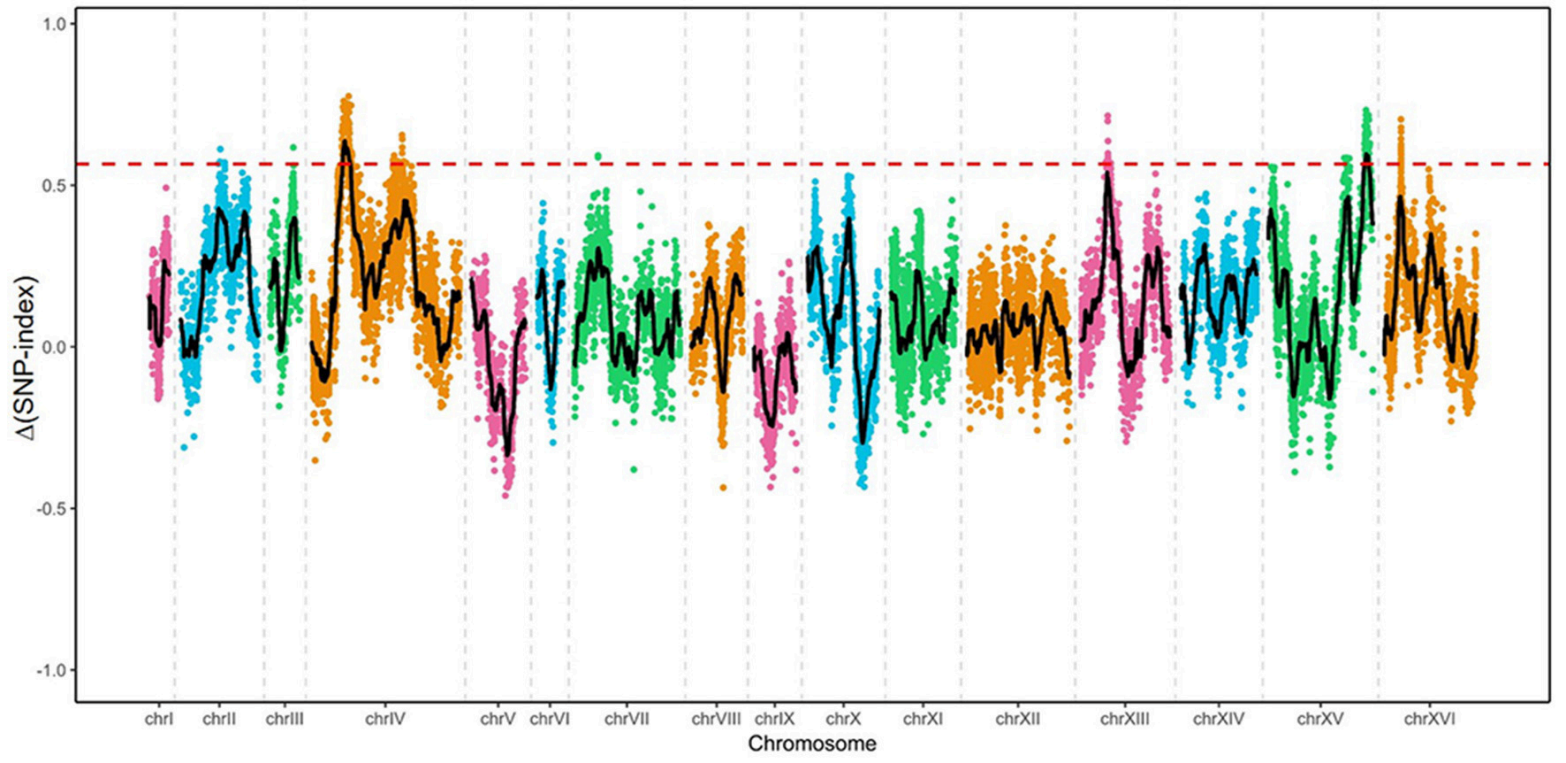
Mapping of the loci involved in near-freezing temperature tolerance by pooled-segregant whole-genome sequence analysis. The X-axis indicates the chromosomes position; Y-axis indicates the Δ(SNP-index) values. The Δ(SNP-index) was the difference between SNP-index of “High-tolerance pool” and that of “Low-tolerance pool.” Black lines show the average Δ(SNP-index) values, as determined using a sliding window analysis with 50 kb window size and 200 bp increment. Red dotted line shows the threshold (0.5660) of Δ(SNP-index).

Genomic DNA from four samples [ZX11(6), NX9412(4), “High-tolerance pool” and “Low-tolerance pool”] was subjected to whole-genome sequencing analysis using the Illumina HiSeq 4000 platform. The sequence reads from two parent strains and the two pools were fist aligned to the reference genome sequence of S288c, to identify SNPs. A total number of 14,619 highly credible SNPs between ZX11(6) and NX9(4) was selected for QTL analysis, which had been quality filtered. Next, the Δ(SNP-index) were calculated and a sliding window analysis with 50 kb window size and 200 bp increment was used for the calculation of the average Δ(SNP-index) of SNPs located in a certain genomic interval. Δ(SNP-index) graph was plotted by aligning an average Δ(SNP-index) against the position of each sliding window in the genome (Figure 5). The peak regions above the threshold value were defined as the candidate regions. By examining the Δ(SNP-index) graph, identified two genomic regions exhibiting the average Δ(SNP-index) above the threshold value of 0.5660: the region on chromosome IV from 319,200 to 397,400 and the region on chromosome XV from 990,600 to 1031,600. In addition, compared to the region located on chromosome XV with the average Δ(SNP-index) values of 0.5872, the region located on chromosome IV exhibiting higher average Δ(SNP-index) values of 0.6156, was selected for further analysis.

### Validation of the causative genes within chromosome IV QTL

**Figure 6:**
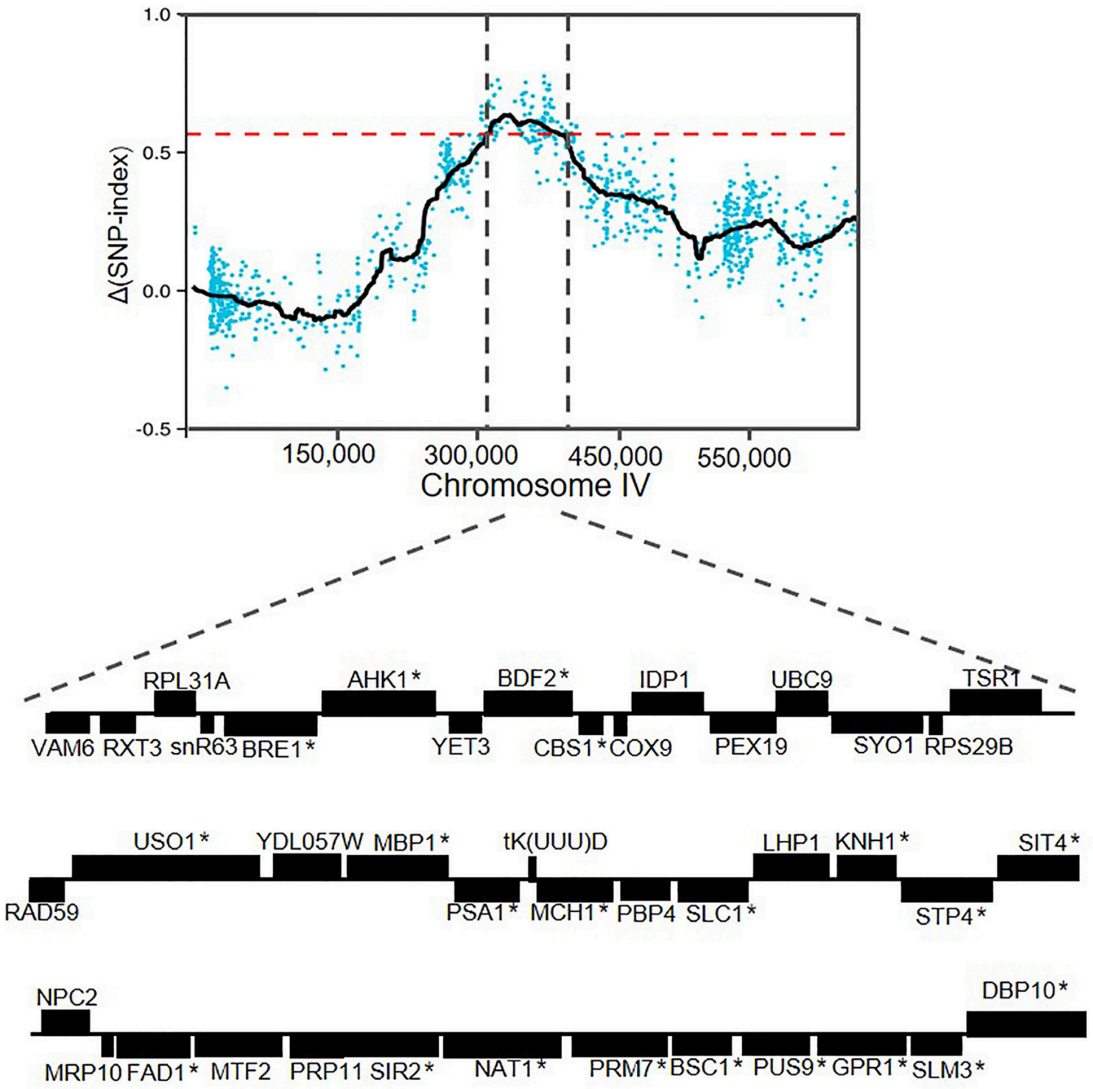
Overview of genes in the QTL on chromosome IV. The region between 319,200 and 397,400 bp, containing 42 genes. The genes containing at least one non-synonymous mutation within the open reading frame were indicated with an asterisk.

The candidate region located on chromosome IV contained 42 genes. A detailed zoom-in of the region and their genes was shown in Figure 6. Detailed analysis of the ZX11(6) sequence of this region revealed that 21 genes contained at least one non-synonymous mutation in the open reading frame compared to the NX9(4) sequence. The presence of these non-synonymous SNPs in the original diploid strain ZX11 and NX9 was tested by sequencing and found that the two diploid strains were heterozygous for these SNPs (data not shown). The occurrence of these non-synonymous mutations in a set of 28 *S. cerevisiae* strains of which the complete genome sequence is known was also checked (Table 2). The results show that most mutations found in the superior ZX11(6) are common in other strains. However, two mutations identified separately in *CBS1* and *NAT1* of ZX11(6) are not found in any one of the 28 sequenced strains and therefore may be unique.

**Table 2:** Occurrence of non-synonymous SNPs in 21 genes in other *S. cerevisiae* strains.

	**BRE1**				**AHK1**		**BDF2**	**CBS1**	**USO1**	**MBP1**	**PSA1**	**MCH1**	**SLC1**	**KNH1**	**STP4**
	**3242**	**3248**	**3253**	**3253**	**3275**	**3291**	**3310**	**3334**	**3496**	**3531**	**3567**	**3604**	**3631**	**3658**	**3680**
	**62**	**92**	**02**	**21**	**40**	**81**	**87**	**67**	**98**	**61**	**71**	**51**	**94**	**65**	**78**
ZX11(6)	T	A	G	C	A	G	G	T	A	G	A	C	T	G	G
NX9(4)	G	G	A	G	G	C	A	C	T	C	T	T	G	A	C
S288C	T	G	A	C	G	G	G	C	A	G	T	C	G	G	C
AWRI1631	T	A	G	C	A	G	A	C	A	G	T	C	T	G	G
AWRI796	T	A	G	C	A	G	A	C	A	G	A	C	T	G	G
BY4741	T	G	A	C	G	G	G	C	A	G	T	C	G	G	C
BY4742	T	G	A	C	G	G	G	C	A	G	T	C	G	G	C
CBS7960	T	G	A	C	G	G	G	C	A	G	T	C	G	G	C
CEN.PK113	T	G	A	C	G	G	G	C	A	G	A	C	G	G	C
DBVPG6044	T	G	A	C	G	G	G	C	A	G	T	C	G	G	C
DBVPG6765	T	A	G	C	A	G	G	C	A	G	T	C	G	G	C
EC1118	T	G	G	C	A	G	G	C	A	G	T	C	T	G	C
SK1	T	G	A	C	G	G	G	C	A	G	T	C	G	G	C
Sigma1278b	T	G	A	C	G	G	G	C	A	C	T	C	G	G	C
W303	T	G	A	C	G	G	G	C	A	G	A	C	G	G	C
YPS128	T	G	A	C	G	G	G	C	A	G	T	C	G	G	C
YJM269	T	G	A	G	G	G	G	C	A	G	T	C	G	G	C
YJM339	T	G	A	C	G	G	G	C	A	G	A	C	G	G	C
YJM789	T	G	A	C	G	G	G	C	A	G	A	C	G	G	C
Kyokai7	T	G	A	C	G	G	G	C	A	G	A	C	G	G	C
T7	T	G	G	C	G	G	G	C	A	G	A	C	T	G	C
LalvinQA23	T	G	A	C	G	G	A	C	T	C	A	T	G	A	C
RM11-1a	T	G	A	C	G	G	G	C	A	G	A	C	G	G	C
UC5	T	A	G	C	A	G	G	C	A	G	A	C	G	G	C
VL3	T	A	G	C	A	G	A	C	A	G	T	C	G	G	C
Vin13	T	G	G	C	A	G	G	C	A	G	T	C	G	G	C
W303	T	A	G	C	A	G	G	C	A	G	T	C	G	G	C
ZTW1	T	G	A	C	G	G	G	C	A	G	A	C	G	G	C
Y12	T	G	A	C	G	G	G	C	A	G	T	C	G	G	C
FostersB	T		G	G	A	G	G		A	C	T	T		G	C
	**SIT4**	**FAD1**	**SIR2**	**NAT1**	**PRM7**			**BSC1**			**PUS9**		**GPR1**	**SLM3**	**DBP10**
	**3701**	**3730**	**3778**	**3802**	**3823**	**3826**	**3828**	**3849**	**3849**	**38547**	**3881**	**3888**	**3907**	**3934**	**3958**
	**01**	**80**	**45**	**52**	**30**	**55**	**29**	**00**	**44**	**3**	**47**	**51**	**56**	**87**	**73**
ZX11(6)	G	C	A	C	G	A	G	T	C	G	G	G	A	G	G
NX9(4)	C	T	G	T	A	G	A	A	T	A	A	A	T	T	A
S288C	G	C	A	T	G	A	G	T	C	A	A	G	A	G	A
AWRI1631	G	C	A	T	G	A	G	T	C	A	G	G	A	G	A
AWRI796	G	C	A	T	G	A	G	T	C	A	G	G	A	G	A
BY4741	G	C	A	T	G	A	G	T	C	A	A	G	A	G	A
BY4742	G	C	A	T	G	A	G	T	C	A	A	G	A	G	A
CBS7960	G	C	A	T	A	G	A	T	C	A	A	G	T	G	A
CEN.PK113	G	C	A	T	G	A	G	T	C	A	A	G	A	G	A
DBVPG6044	G	C	A	T	A	G	G			A	A	G	T	G	A
DBVPG6765	G	C	A	T	G	A	G		C	G	G	G	A	G	G
EC1118	G	C	A	T	G	A	G	T	C	A	A	G	A	G	G
SK1	G	C	A	T	A	G	G			A	A	G	T	G	A
Sigma1278b	G	T	A	T	G	A	G			A	A	G	T	G	A
W303	G	C	A	T	A	G	G	A		A	A	G	T	G	A
YPS128	G	C	A	T	A	G	A		T	A	A	G	T	G	A
YJM269	G	C	A	T	A	G	A	A	C	A	G	G	A	G	A
YJM339	G	C	A	T	A	G	A	A	C	A	A	G	T	G	A
YJM789	G	C	A	T	G	A	G	A	C	A	G	G	A	G	A
Kyokai7	G	C	A	T	A	G	A	T	T	A	A	G	T	G	A
T7	G	C	A	T	G	A	G	T	C	A	A	G	A	G	G
LalvinQA23	C	C	-	T	A	G	A	T	T	A	A	A	A	G	G
RM11-1a	G	C	A	T	G	A	G	T	C	A	A	G	A	G	A
UC5	G	C	A	T	G	A	G	T	C	A	G	G	A	G	G
VL3	G	C	A	T	G	A	G	T	C	G	A	G	A	G	G
Vin13	G	C	A	T	G	A	G	T	C	A	G	G	A	G	A
W303	G	C	A	T	G	A	G	T	C	A	A	G	A	G	G
ZTW1	G	C	A	T	G	A	G	T	C	A	A	G	A	G	A
Y12	G	C	A	T	A	G	A	A	T	A	G	G	T	G	A
FostersB	G	C	A	T	A	G	A	T	T	G	A	G	T	G	A

**Figure 7:**
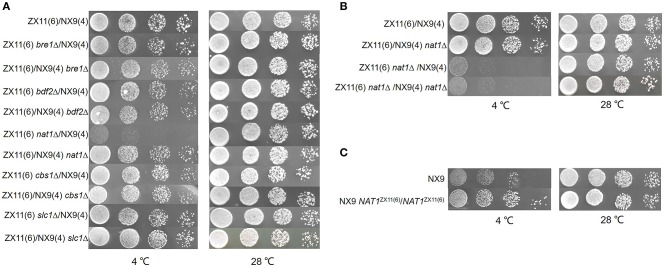
Identification of the causative gene *NAT1*. **(A)** RHA results of the five selected genes. Spot growth assay on the hemizygous diploids for *BRE1, BDF2, NAT1, CBS1*, and *SLC1* were shown. The reciprocal hemizygote containing the ZX11(6) allele grew better than the one presenting the allele of NX9(4) at 4°C. **(B)** Effect of *NAT1* deletion on near-freezing temperature tolerance. The *NAT1* was deleted in the hybrid diploid ZX11(6)/NX9(4) background. ZX11(6) *nat1*Δ/NX9(4) *nat1*Δ showed the same growth as ZX11(6) *nat1*Δ/NX9(4) at 4°C and ZX11(6)/NX9(4) *nat1*Δ showed the same growth as ZX11(6)/NX9(4) at 4°C. **(C)** *NAT1* allele replacement in strain NX9. The both *NAT1* alleles in the original sensitive parent strain (NX9) was replaced by the *NAT1*^ZX11(6)^ alleles. Allelic exchange of *NAT1*^ZX11(6)^ led to a remarkable positive impact on the growth at 4°C.

The protein function of candidate genes presenting such polymorphisms was also considered. Five potential candidate genes (*BRE1, BDF2, NAT1, CBS1*, and *SLC1*) within the QTL were selected to investigate their effect on the near-freezing temperature tolerance using reciprocal hemizygosity analysis. The gene function and the protein sequence changes in the parental strain are shown in Table 3. For validation of the genes, five pairs of hemizygous diploid ZX11(6)/NX9(4) hybrid strains were constructed, in which each pair retained a single copy of the superior [ZX11(6)] or inferior parent [NX9(4)] allele of *BRE1, BDF2, NAT1, CBS1*, or *SLC1* respectively, while the other copy of the gene was deleted. From the results of RHA (Figure 7A), the hemizygotes for *NAT1* significantly affected the growth at near-freezing temperature (4°C), while had no effect on the growth at 28°C. The reciprocal hemizygote containing the ZX11(6) allele grew better than the one presenting the allele of NX9(4) at 4°C. However, compared to the control, hemizygous diploid strains of the other four genes had no effect on the growth at near-freezing temperature (4°C) and the optimum temperature (28°C). The results indicated that *NAT1* is probably a causative gene, involved in the near-freezing temperature tolerance in *S. cerevisiae*. Compared to the NX9(4) allele, the *NAT1* allele of ZX11(6) contains one non-synonymous point mutation, within its coding sequence (position 380,256 bp of chromosome IV), changing the amino acid sequence at position 396 from phenylalanine to serine. Moreover, we further confirmed the relevance of *NAT1* by demonstrating that *NAT1* deletion reduced near-freezing temperature tolerance in the hybrid diploid ZX11(6)/NX9(4) background (Figure 7B). We constructed a ZX11(6)/NX9(4) hybrid diploid strain with both *NAT1* deleted and compared its tolerance phenotype with that of ZX11(6)/NX9(4) with its original *NAT1* alleles. Since ZX11(6) *nat1*Δ/NX9(4) *nat1*Δ showed the same growth as ZX11(6) *nat1*Δ/NX9(4) at 4°C and since ZX11(6)/NX9(4) *nat1*Δ showed the same growth as ZX11(6)/NX9(4) at 4°C, the *NAT1*_NX9(4)_ allele behaves as a loss of function allele, at least in our conditions and in the hybrid background.

**Table 3:** The candidate genes were selected according to their function and non-synonymous SNPs in the parental strain sequences.

**Gene**	**Function**	**Changes in protein sequence**
*BRE1*	E3 ubiquitin-protein ligase, involved in double strand break repair, histone ubiquitination, chromatin silencing	ZX11(6): E283G, K357B, E420K NX9(4): L277V, K357B, S630A
*BDF2*	Protein involved in transcription initiation; acts at TATA-containing promoters; associates with the basal transcription factor TFIID	ZX11(6): D340E NX9(4): A22T, K317E
*NAT1*	Subunit of protein N-terminal acetyltransferase NatA; involved in N-terminal protein amino acid acetylation	ZX11(6): F396S
*CBS1*	Mitochondrial translational activator of the COB mRNA; membrane protein that interacts with translating ribosomes, acts on the COB mRNA 5′-untranslated leader	ZX11(6): T155I
*SLC1*	1-acyl-sn-glycerol-3-phosphate acyltransferase; involved in glycerophospholipid biosynthetic process	ZX11(6): R130S

### Improvement of the near-freezing adaption in strain NX9

The both *NAT 1* alleles in the original sensitive parent strain (NX9) was replaced by the *NAT1*^ZX11(6)^ alleles using homologous recombination. A NX9 *nat1*Δ/*nat1*Δ strain and a NX9 *NAT1*^ZX11(6)^/*NAT1*^ZX11(6)^ train were constructed and evaluated for their ability to grow at 4°C and 28°C by spot assay on YPD agar plates. The results were shown in Figure 7C. The NX9 *NAT1*^ZX11(6)^/ *NAT1*^ZX11(6)^ strain grew much better than the NX9 strain at 4°C, indicating that allelic exchange of *NAT1*^ZX11(6)^ led to a remarkable positive impact on the growth at 4°C. The results confirm that the superior allele *NAT1*^ZX11(6)^ could be used to genetically improve the near-freezing adaption of industrial yeast strains.

## Discussion

Microorganisms that face severe low temperatures in their natural habitat must be equipped with cellular mechanisms to respond and adapt to the change. For yeast, the cold shock adaptation at low but still permissive temperatures (10–18°C) has been quite extensively investigated. However, the cold shock response at the temperature below 10°C has only caught a little attention of researchers.

To date, several studies have been carried out to identify the genes affecting the tolerance to near-freeze temperature in *S. cerevisiae* by several approaches, including transcriptomic and other high-throughput strategies. Genome-wide expression analysis showed that at 4°C, a number of genes involved in glycogen (such as *GAC1, GLC3*, and *GPH1*), glycerol (i.e., *GPD1* and *STL1*) and trehalose (i.e., *TPS1* and *TPS2*) biosynthesis are significantly upregulated (Homma et al., 2003; Schade et al., 2004; Murata et al., 2006; Panadero et al., 2006). The induction of heat-shock genes by near-freezing temperatures is of interest to researchers and has been reported by several studies. When yeast cells are cultured at 4°C, several HSPs (*HSP12, HSP42, HSP104*, and *SSA4*) are induced, suggesting that induction of these genes may be necessary for adaptation to cold resistance (Homma et al., 2003; Kandror et al., 2004; Becerra et al., 2010). These candidate-gene identification approaches however, are quite limited to explain the genetic basis of the trait and its application potentiality. As with many stress-resistance traits in yeast, the near-freezing tolerance is a complex trait dominated by multiple quantitative trait loci (QTLs) and influenced by environment. The use of QTL mapping to identify the genes underlying this phenotype should give us a deeper understanding of the genetic basis of variability of near-freezing temperature tolerance.

In this work, to investigate the genetic basis, we constructed a recombined F_2_ segregant population by crossing two Chinese indigenous *S. cerevisiae* strains with a divergent phenotype, used the BSA approach to identify major QTL involved in the near-freezing temperature tolerance by analyzing the SNP-index of two pools. BSA is an efficient and rapid approach for detection of major QTLs by genotyping two DNA pools from the offspring with contrasting phenotypes (Pomraning et al., 2011), and has been commonly used to determine the genetic basis of many complex traits in yeast (Hubmann et al., 2013b; Pais et al., 2013; Brice et al., 2014; Carvalho et al., 2017). In our study, two hot genomic regions were identified by genome-wide comparison of Δ(SNP-index). The region located on chromosome IV exhibiting highest average Δ(SNP-index) values, was selected for further analysis. Although this region contains a high number of genes (42 genes), based on the protein function analysis as well as non-synonymous mutations information in these genes, five potential genes were selected to identified to investigate their effect on the near-freezing temperature tolerance. And finally, we have successfully identified a gene *NAT1*, associated with the near-freezing temperature tolerance by RHA and gene deletion. This way is less costly and more efficient than fine mapping by scoring selected SNPs by allele-specific PCR in a number of individuals.

The results revealed the gene *NAT1*, whose allelic variation influenced the tolerance to low-temperature. Introduction of the superior allele *NAT1*^*ZX*11(6)^ in original sensitive diploid strain (NX9) improved its near-freezing temperature tolerance. This confirms that the superior allele *NAT1*^*ZX*11(6)^ could be used to genetically improve the near-freezing adaption of industrial yeast strains in the future. The protein Nat1p is one of three subunits (Nat1p, Ard1p, and Nat5p) of N-terminal acetyltransferase NatA, responsible for anchoring Ard1p to the ribosome, contributes to NatA's enzymatic activity (Mullen et al., 1989; Gautschi et al., 2003). Some studies reported that the deletion of *NAT1* can result in a phenotype of temperature sensitivity (Bogdan et al., 1999; Arnesen et al., 2009). Arnesen et al. (2009) reported the growth ability at low-temperature of *nat1*Δ strain was significantly decreased. But their research conditions were at 15°C, which is a low but still permissive temperature. In our study, we found that the gene *NAT1* was associated with the near-freezing temperature (4°C) tolerance. Hence, the result of our study not only has brought something new to the study on the near-freezing stress tolerance in yeast but also has complemented the functional research of *NAT1*.

So far, no reported study has explored the genetic determinants of the near-freezing tolerance in *S. cerevisiae* using QTL mapping approach. Our study is the first to use QTL mapping approach. This will further assist us to understand the molecular mechanism underlying this complex trait.

## Author contributions

LF, YL, and ST conceived and designed the experimental program. LF, HJ, and YQ performed all experiments. LF and HJ analyzed the results. LF and YL drafted the manuscript. YS provided some suggestions for the manuscript. All authors read and approved the final manuscript.

### Conflict of interest statement

The authors declare that the research was conducted in the absence of any commercial or financial relationships that could be construed as a potential conflict of interest.
