# Supplementary material for: Rapid Identification of Major QTLS Associated With Near- Freezing Temperature Tolerance in Saccharomyces cerevisiae
Source: Front Microbiol. 2018 Sep 11;9:2110. doi: 10.3389/fmicb.2018.02110 (PMC6141824; doi:10.3389/fmicb.2018.02110)
Supplement: Supplementary file 1 [file Table_1.DOCX]

Supplementary Material

**Rapid identification of major QTL_S_ associated with near-freezing temperature tolerance in** ***Saccharomyces cerevisiae***

Li Feng^1^, He Jia^1^, Yi Qin^1^, Yuyang Song^1^, Shiheng Tao^2,^ *, Yanlin Liu^1,^ *

*** Correspondence:**Yanlin Liu
ylliu@nwsuaf.edu.cn

Shiheng Tao

Shihengt@nwafu.edu.cn

# Supplementary Table S1. 50 wild diploid *S. cerevisiae* strains used in this study.

| **Strain** | **Geographical origin** |
| --- | --- |
| XJ1 | Xinjiang wine production region |
| XJ2 | Xinjiang wine production region |
| XJ3 | Xinjiang wine production region |
| XJ4 | Xinjiang wine production region |
| XJ5 | Xinjiang wine production region |
| XJ6 | Xinjiang wine production region |
| XJ7 | Xinjiang wine production region |
| XJ8 | Xinjiang wine production region |
| XJ9 | Xinjiang wine production region |
| IM1 | Inner Mongolia wine production region |
| IM2 | Inner Mongolia wine production region |
| IM3 | Inner Mongolia wine production region |
| IM4 | Inner Mongolia wine production region |
| IM5 | Inner Mongolia wine production region |
| IM6 | Inner Mongolia wine production region |
| IM7 | Inner Mongolia wine production region |
| ZX1 | Gansu wine production region |
| ZX2 | Gansu wine production region |
| ZX3 | Gansu wine production region |
| ZX4 | Gansu wine production region |
| ZX5 | Gansu wine production region |
| ZX6 | Gansu wine production region |
| ZX7 | Gansu wine production region |
| ZX8 | Gansu wine production region |
| ZX9 | Gansu wine production region |
| ZX10 | Gansu wine production region |
| ZX11 | Gansu wine production region |
| GS1 | Gansu wine production region |
| GS2 | Gansu wine production region |
| GS3 | Gansu wine production region |
| GS4 | Gansu wine production region |
| GS5 | Gansu wine production region |
| GS6 | Gansu wine production region |
| GS7 | Gansu wine production region |
| NX1 | Ningxia wine production region |
| NX2 | Ningxia wine production region |
| NX3 | Ningxia wine production region |
| NX4 | Ningxia wine production region |
| NX5 | Ningxia wine production region |
| NX6 | Ningxia wine production region |
| NX7 | Ningxia wine production region |
| NX8 | Ningxia wine production region |
| NX9 | Ningxia wine production region |
| SAX1 | Shaanxi wine production region |
| SAX2 | Shaanxi wine production region |
| SAX3 | Shaanxi wine production region |
| SAX4 | Shaanxi wine production region |
| SAX5 | Shaanxi wine production region |
| SAX6 | Shaanxi wine production region |
| SAX7 | Shaanxi wine production region |
